# Promoter Methylation status of HIN-1 associated with outcomes of ovarian clear cell adenocarcinoma

**DOI:** 10.1186/1476-4598-11-53

**Published:** 2012-08-08

**Authors:** Chih-Ming Ho, Chi-Jung Huang, Chia-Yen Huang, Yih-Yiing Wu, Shwu-Fen Chang, Wen-Fang Cheng

**Affiliations:** 1 Gynecologic Cancer Center, Department of Obstetrics and Gynecology, Cathay General Hospital, Taipei, Taiwan; 2 Department of Medical Research, Sijhih Cathay General Hospital, New Taipei City, Taiwan; 3 School of Medicine, Fu Jen Catholic University, Hsinchuang, New Taipei City, Taiwan; 4 Department of Obstetrics and Gynecology, School of Medicine, Taipei Medical University, Taipei, Taiwan; 5 Department of Pathology, Sijhih Cathay General Hospital, New Taipei City, Taiwan; 6 Graduate Institute of Medical Sciences, School of Medicine, Taipei Medical University, Taipei, Taiwan; 7 Graduate Institute of Oncology, Graduate Institute of Clinical Medicine, Department of Obstetrics and Gynecology, College of Medicine, National Taiwan University, Taipei, Taiwan

**Keywords:** Ovarian clear cell adenocarcinoma, Methylation-specific multiplex ligation-dependent probe amplification, HIN-1, CACNA1A

## Abstract

**Background:**

This study is to analyze promoter methylation of various tumor suppressor genes in different types of ovarian carcinoma and to identify potential therapeutic targets of ovarian clear cell adenocarcinoma (OCCA).

**Materials and methods:**

The promoter methylation statuses of 40 genes in primary ovarian carcinomas including 47 clear- and 63 non-clear-cell type tissues, 6 OCCA cell lines, 29 benign ovarian endometriotic cysts, and 31 normal controls were analyzed by methylation-specific multiplex ligation-dependent probe amplification (MS-MLPA). The MS-MLPA results were correlated with clinicopathological features and outcomes of 47 OCCA patients. Functions of the target genes were further explored by Western Blot Analysis, apoptosis assay, and caspase-3/7 activity analysis.

**Results:**

Frequencies of methylated RASSF1A, CDH13, CACNA1A, HIN-1, and sFRP5 genes in OCCA tissues were significantly higher than those in non-OCCA cancerous tissues and benign endometriotic cysts**.** The expected OS for patients with methylated promoters of HIN-1 was significantly worse than those for patients without methylated HIN-1 (30% vs. 62%, *p* = 0.002). The HIN-1 gene was over-expressed in ES2 cells, a significant reduction in cell growth and induction of apoptosis, and increasing paclitaxel sensitivity by reducing phosphorylation of Akt were observed.

**Conclusions:**

Methylation of HIN-1 promoter is a novel epigenetic biomarker associated with poor outcomes in OCCA patients. Ectopic expression of the HIN-1 gene increased paclitaxel sensitivity which is partly through Akt pathway.

## Introduction

Ovarian clear cell adenocarcinoma (OCCA) is recognized as a distinct histological type of cancer, and its frequency among epithelial ovarian cancers is estimated to be less than 10% [1,2]. However, the overall frequency of OCCA is higher in Taiwan at about 15-20% of epithelial ovarian cancers [3]. OCCA clinically differs from other epithelial ovarian cancers because of its *de novo* resistance to platinum-based chemotherapies, and it shows a poor prognosis [3-5].

The molecular pathogenesis of OCCA is still unclear and needs to be elucidated to improve patient outcomes. However, hepatocyte nuclear factor-1β is upregulated in OCCA cells compared to non-OCCA cells, and was reported to be essential for the survival of patients [6]. Higher p21 and cyclin E with lower TP53 and cyclin A levels were detected in OCCA compared to other epithelium ovarian cancers, and they are thought to be involved in the carcinogenesis of OCCA [7]. Silencing of Wilms tumor suppressor 1 sense and antisense genes by promoter methylation in OCCA revealed the epigenetic involvement of OCCA in carcinogenesis as distinguished from ovarian serous adenocarcinoma [8]. Recently, the high percentage of promoter methylation of the *sFRP5* gene in OCCA indicated its importance in the development of OCCA and is a potentially useful marker for prognoses and treatment targeting of OCCA [9]. Neither PTEN promoter methylation nor loss of homozygosity (LOH) at the 10q23 locus was significantly related to PTEN inactivation, which is often detected in OCCA [10]. Activating mutations in the PIK3CA gene [11] and genomic amplification of chr20q13.2 [12] are also common genetic alterations identified with OCCA. Recently, mutations at PPP2R1A and ARID1A were found, and it was suggested that aberrant chromatin remodeling may contribute to the pathogenesis of OCCA, indicating that epigenetic changes in cancer cells may occur through specific modifications of chromatin proteins [13].

Hypermethylation of CpG islands within the regulatory region of tumor suppressor genes (TSGs) is one of the earliest and most frequent alterations; it results in gene silencing and confers a growth advantage on tumor cells [14]. Distinct patterns of DNA methylation among different tumors may be a useful signature for diagnosis and prognosis [15]. Loss of sFRP5 was recently reported to be an aberrant molecular event in OCCA and a possible prognostic marker [9]. Cellular events affected by epigenetic alterations include DNA repair, cell cycling control, adherence, apoptosis, and detoxification [16]. Thus, a complicated epigenetic network is thought to be involved in OCCA carcinogenesis. We hereby hypothesized that additional cancer-related genes with aberrant methylation modified promoters possibly contribute to the pathogenesis and progression of OCCA. As the number of methylated genes revealed in cancer is increasing, sensitive and robust multiplex methods for detecting the methylation status of promoter regions are desirable. Therefore, a methylation-specific multiplex ligation-dependent probe amplification (MS-MLPA) analysis was applied to determine the TSG promoter methylation profile of OCCA.

## Materials and methods

### Cell lines and cultures

OCCA cell lines including HAC-2, KK, RMG-I, RMG-II, and ES-2 cells, and two immortalized cell lines, OSE2a and OSE2b-2 (tumorigenic), were cultured and maintained as described previously [10]. TOV21G was obtained from American Type Culture Collection (ATCC) and maintained in MCDB 105/medium 199 supplemented with 10% heat-inactivated fetal bovine serum.

### Patients and specimens

Tissue samples were obtained from surgical specimens with the informed consent of patients at Cathay General Hospital (CGH) after this project being approved by the Institutional Review Board of the hospital. Tissues were taken only from cancerous regions during surgery were immediately frozen at −80°C until analysis and each sample was confirmed pathologically to have high neoplastic cellularity (> 66%).

In total, 110 primary human epithelial ovarian carcinoma samples, comprising 47 OCCA and 63 non-OCCA tissues, and 29 benign endometriotic cysts were collected between 1994 and 2005. The histologic grading was based on International Union against Cancer criteria, while staging was according to the criteria set by the International Federation of Gynecology and Obstetrics [17].

Preexisting clinical information, including age, menopausal status, treatment history, surgical findings during debulking, recurrence status, and survivorship was collected from clinical and operative notes and the discharge summary that were deposited in a centralized database. The maximal diameter of the residual tumor during surgery was also recorded. Optimal debulking surgery was defined as the maximal diameter of the residual tumor of < 1 cm or otherwise defined as suboptimal debulking surgery. Patients received regular follow-up after completion of treatment. Computed tomography (CT) or magnetic resonance imaging (MRI) was performed when recurrence was suspected. Abnormal results of imaging studies, aspiration cytology from ascites, elevated tumor markers such as CA125 (≥ 2-fold of the upper normal limit) on two consecutive tests within a 2-week interval, or tissue proven from biopsy, if possible, was defined as recurrence [18]. Progression-free survival was measured as the period from the operation to the date of confirmed recurrence or disease progression, or the date of the investigators’ last note of a disease-free status.

### **Methylation-specific multiplex ligation-dependent probe amplification** (**MS-MLPA)**

MS-MLPA with SALSA MLPA kits such as ME001B and ME003-A1 (MRC-Holland, Amsterdam, the Netherlands) are commercially available kits which were applied with specifically designed probe sets to detect the methylation status in the promoter regions of 40 TSGs **(**Table 1**)**. These 40 TSG were reported to be associated with cancer in the literature. All control probes that were not influenced by *Hha* I digestion were included. The MS-MLPA reaction was performed according to the MRC-Holland’s user guide with some modifications [19]. In brief, 300 ng of genomic DNA from respective tissue samples or cell lines was used. The MS-MLPA probes were hybridized to denatured genomic DNA for 16 h at 60°C, then divided into two aliquots. One aliquot was ligated and digested with the methylation-sensitive *Hha* I restriction enzyme, and the other was ligated but without *Hha* I digestion. Ligated probes were amplified by a polymerase chain reaction (PCR) using fluorescently labeled primers to produce a uniquely sized product. Following restriction digestion, probe-targeting sequences containing unmethylated *Hha* I sites could not be amplified. PCR products were examined by capillary electrophoresis on an ABI 3100 genetic analyzer, using GENESCAN software (version 3.5.1, Applied Biosystems). Peak identification and values corresponding to peak size in base pairs (bp), and peak areas were further processed to obtain data, which were then normalized to control DNA and extracted using Coffalyser software (version 9.4, MRC-Holland). For the hypermethylation analysis, the “relative peak value” or the “probe fraction” of the ligation-digestion sample was divided by the “relative peak value” of the corresponding undigested ligation sample, resulting in a “methylation-ratio” (M-ratio). Aberrant methylation was scored when the calculated M-ratio was ≥ 0.30, corresponding to 30% methylated DNA. Values of 0.00 ~ 0.29 of the M-ratio were interpreted as the absence of hypermethylation. When the percentage ratio was < 30%, it was relegated to background noise. M-ratios of 0.30 ~ 0.49 were recognized as mild hypermethylation, 0.50 ~ 0.69 as moderate hypermethylation, and > 0.70 as extensive hypermethylation. For reaction-targeted genes with more than one probe, M-ratios were independently calculated for the methylation analysis, and we also used the previously described mathematical algorithm cutoff ratio for a peak height of ≥ 30% [20,21].

**Table 1:** Genes represented by probe mixtures (ME001B and ME003-A1)

**ME001B**			**ME003-A1**		
**Gene**	**Chromosonal position**	**UniGene**	**Gene**	**Chromosomal position**	**UniGene**
APC	5q21	Hs.158932	BCL2	18q21.3	Hs.150749
ATM	11q23	Hs.367437	BNIP3	10q26.3	Hs.144873
BRCA1	17q21	Hs.194143	CACNA1A	19p13.2	Hs.501632
BRCA2	13q12	Hs.34012	CACNA1G	17q21.33	Hs.591169
CASP8	2q33-q34	Hs.599762	CCND2**	12p13.3	Hs.376071
CD44	11p13	Hs.502328	DLC1**	8p22	Hs.134296
CDG13	16q24.2	Hs.654386	H2AFX**	11q23.3	Hs.477879
CDKN1B	12p13.1	Hs.238990	ID4**	6p22.3	Hs.519601
CDKN2A	9p21	Hs.238990	PRDM2	1p36.21	Hs.371823
CDKN2B	9p21	Hs.72901	RARB*	3p24	Hs.654490
CHFR	12q24.33	Hs.656770	RUNX3	1p36.11	Hs.170019
DAPK1	9q34.1	Hs.380277	SCG3A1**	5q35	Hs.62492
ESR1	6q25.1	Hs.208124	SFRP4**	7p14.1	Hs.658169
FHIT	3p14.2	Hs.715588	SFRP5**	10q24.1	Hs.279565
GSTP1	11q13	Hs.523836	SMARCA3**	3q25.1	Hs.3068
HIC1	17p13.3	Hs.72956	TGIF	18p11.31	Hs.373550
IGSF4	11q23	Hs.370510	TIMP3	22q12.3	Hs.644633
MLH1	3p21.3	Hs.195364	TWIST1	7p21.2	Hs.66744
PTEN	10q23.31	Hs.500466			
RASSF1	3p21.3	Hs.476270			Hs.
TP73	1p36	Hs.697294			
VHL	3p26-p25	Hs.517792			

### Sodium bisulfite treatment, sequencing, and methylation specific-PCR analyses of the genome

Genomic DNA was isolated using a Genomic DNA kit (Geneaid Biotech, Bade City, Taiwan), converted with sodium bisulfite in the CpGenome DNA modification kit (Millipore, MA, USA), purified, and then amplified by a PCR with DNA polymerase (ThermoHotStart 2X Gold PCR Master mix; Applied Biosystems) and HIN-1-specific primers. The primer sequences for methylated *HIN-1* were 5’-GAAGTTTCGTGGTTTTGTTCG-3’ (forward) and 5’-AAAACCTAAAATCCACGATCGAC-3’ (reverse); and the primer sets for unmethylated HIN-1 were 5’-TAAGAAGTTTTGTGGTTTTGTTTGG-3’ (forward) and 5’-AAAAAACCTAAAATCCACAATCAAC-3’ (reverse). Bisulfite-modified, *Sss* I (New England Biolabs, MA)-treated normal lymphocyte DNA served as the methylated control, and bisulfite-treated normal lymphocyte DNA was the unmethylated control. PCR products were analyzed on 3% agarose gels. The methylation specific-PCR in a final volume of 20 μl was performed under the following conditions: 95°C for 10 min, followed by 40 cycles of 95°C for 30 s, 62°C for 30 s, and 72°C 40 s; with a final extension at 72°C for 10 min and holding at 4°C. PCR products were purified and then directly sequenced using an Applied Biosystems ABI automated DNA sequencer.

### 5-Aza-2-deoxycytidine treatment and reverse-transcription real-time quantitative (RT-qPCR) analysis

Cells were treated with 10 μM of 5-aza-2-deoxycytidine (5-aza-2-dC; Sigma, St. Louis, MO, USA), and renewed every 24 h. An RT-qPCR analysis was used to measure HIN-1 messenger RNA in response to treatment. Constitutively expressed GAPDH was used as an internal control. The qPCR was performed in an ABI Prism 7300 Sequence Detection System (Applied Biosystems) with Taqman Gene Expression Assay Hs00369360g1 and primers as described above. The conditions were as follows: 2 min at 50°C, 10 min at 95°C, and then 40 cycles of 95°C for 15 s and 60°C for 1 min. The interpolated number (Ct) of cycles to reach a fixed threshold above the background noise was used to quantify amplification.

### Western blot analysis

Cell lysates were prepared using a protein extraction solution (iNtRON Biotechnology, Kyunggi-do, Korea), separated by sodium dodecyl sulfate polyacrylamide gel electrophoresis (SDS-PAGE) (12%) and transferred to a polyvinylidene difluoride (PVDF) membrane. The membrane was blocked in TBS blocking buffer (0.2% Tween 20 and 3% bovine serum albumin) and incubated with the indicated antibodies for 1 h. After washing, a peroxidase-conjugated rabbit-anti-goat immunoglobulin G was applied, and the bound antibodies were visualized by development with NBT/BCIP as the chromogenes. The GST-HIN-1 fusion protein was used as a positive control. An affinity-purified anti-human β-actin rabbit polyclonal antibody (Santa Cruz Biotechnology, CA) was applied to normalize the signals generated by the anti-*Hin*-1 antibody (Santa Cruz Biotechnology).

### Immunohistochemistry (IHC)

Formalin-fixed, paraffin-embedded specimens were sliced by a microtome at a thickness of 1 ~ 3 μm and placed on coated slides. Tissue slides were then incubated with a purified goat polyclonal antibody of UGRP2 (S-15) (Santa Cruz Biotechnology) using a Thermo Scientific Autostainer 360 (Thermo Fisher Scientific Inc, CA). The expression was scored as 0, 1, 2, or 3 according to the intensity. Tissues with > 10% of neoplastic cells and expressing a score of 2 ~ 3 intensity were recognized as positive. Percentages of each score in positive tissues were further recorded. A pathologist not involved in the present study evaluated the immunostaining under blinded conditions.

### Plasmid DNA construction, preparation, and transfection

HIN-1 full-length complementary DNA (cDNA) was amplified by a PCR using human placenta cDNA as the template and primers of 5′-GAATTCATGAAGCTCGCCGCCCTCC-3′ and 5′-CTCGAGTCAGCCAAACACTGTCAGGG-3′. The amplified product was cloned into *EcoR* I/*Xho I* sites of pcDNA3.1 (Invitrogen), and then confirmed by sequencing.

ES-2 cells (3x10^5^ cells/well of 6-well plate) were transfected with an HIN-1 expression plasmid using FuGENE HD (Roche Applied Science) according to the manufacturer’s protocol. Transfected ES-2 cells were selected with 400 μg/ml G418 (Invitrogen). Stably transfected cells with the Hin-1-expression plasmid (ES-2-Hin-1) or with an empty vector (ES-2-PCDNA3.1) were maintained with 200 μg/ml of G418.

### Drug treatment and cell growth assays

ES-2-Hin-1 or ES-2-PCDNA3.1 cells were plated at 1x10^4^ cells/well in 6-well plates for 24 h, and treated with different concentrations of paclitaxel for the indicated times. The culture medium was changed every 2 days. The number of viable cells after paclitaxel treatment was counted with a trypan blue dye exclusion assay in a hemocytometer.

### Annexin V apoptosis assay and caspase-3/7 activity analysis

Apoptosis-positive cells were analyzed with an FITC Annexin V apoptosis detection kit I (BD Biosciences) according to the manufacturer’s protocol with minor modifications. Briefly, ES2 cells were seeded in 12-well chamber slides (ibidi, Martinsried, Germany) at a density of 7500 cells/well for 24 h and treated with 25 nM taxol or 4 μM cisplatin for 6 h. Each well was washed with wash buffer (20 mM Tris (pH 7.4) 150 mM NaCl, and 1 mM CaCl_2_) following a 15-min incubation in binding buffer containing Annexin V-FITC and propidium iodide (PI) at room temperature in the dark. Afterwards, apoptotic images were caught on a fluorescence microscope (Nikon ECLIPSE 80i). Additional evidence for the occurrence of apoptosis was quantitatively determined by the activity of cellular caspase-3/7 using Caspase-Glo 3/7 assays (Promega) according to the manufacturer’s instructions. Briefly, ES2 cells were seeded and treated with paclitaxel as described above. After 24 h, cells were lysed and luminogenic substrates specific for the caspase species were added. Light emission was measured in a luminometer (Berthold Technologies, Wildbad, Germany).

### Western blot analysis to detect phosphorylated AKT

ES-2-Hin-1 or ES-2-PCDNA3.1 cells were plated overnight and starved in serum free medium for 24 hours. Then the cells were cultured in complete medium with 15nM paclitaxel for indicated intervals. Western blot analysis was performed as described above. Briefly, three antibodies (anti- AKT, anti-phospho-AKTThr308, and anti-phospho-AKTSer473) (Cell Signaling, Beverly, MA) were used.

### Statistical analysis

Statistical analyses were performed using the SPSS statistical package (SPSS 16.0.1 for Windows 2008, Chicago, IL). Frequencies of promoter methylation of 40 genes were compared using a Chi-squared test. Survival curves were generated using the Kaplan-Meier method. Differences in survival curves were calculated using the log-rank test. Cox’s univariate and multivariate regression analyses were used to evaluate prognostic factors for survival. A *p* value < 0.05 was regarded as statistical significance.

## Results

The mean age of the 47 OCCA patients was 50 (range, 32 ~ 66) years. The distribution of stages was 4 at stage IA, 13 at IC, 2 at IIA, 4 at IIC, 1 at IIIA, 18 at IIIC, and 5 at IV. The percentages of optimal and suboptimal debulking surgeries were 72.3% and 27.7%. Twenty-eight (60.9%) of them had preoperative CA125 serum level higher than 500 IU/ml.

All OCCA patients underwent staging laparotomy and initial cytoreductive surgery followed by both paclitaxel-platinum-based chemotherapy. The median number of cycles was six (range: 4–9 cycles). Clinical response was assessed in patients with clinically measurable disease, according to WHO criteria, or assessed in patients with nonmeasurable disease, according to normal physical examinations, computed tomography of the abdomen or pelvis and chest X-ray, and CA125 <35 U/ml. Cytoreduction was optimal (residual disease less than 1 cm) in 13 of the 24 patients (54%) with stage III and IV OCCA, and was suboptimal in 11 patients (46%). For patients with stage III and IV OCCA, 58.3% (14/24) had complete or partial response to chemotherapy. However, patients with stage III and IV OCCA exhibited a 54% (13/24) high rate of platinum resistance (recurrence occurred in 6 months after completing chemotherapy) or refractory (disease progression during chemotherapy).

### MS-MLPA profiling of TSG promoter methylation in OCCA cell lines

We first evaluated TSG promoter methylation by MS-MLPA on the OCCA cell lines― ES2, KK, HAC-2, RMG-1, RMG-2, and TOV21G. Promoters of RASSFIA1, HIN-1, ID4, sFRP4, sFRP5, CCND2, CDH13, and CACNA1A genes were found to be methylated in at least two of the six analyzed OCCA cell lines.

These results suggest that these genes could be epigenetic candidates to distinguish aberrant methylation in human OCCA tissues.

### MS-MLPA profiles of TSG promoter methylation in OCCA tissues

We further evaluated TSG promoter methylation of OCCA tissues by MS-MLPA. Eight of the 40 TSG promoters showed significantly higher frequencies of hypermethylation in OCCA tissues compared to benign ovarian cysts **(**Table 2**)**. The most frequently hypermethylated genes in OCCA tissues analyzed by MS-MLPA in order were RASSFIA (77%), CCND2 (51%), CDH13 (47%), CACNA1A (43%), HIN-1 (40%), sFRP5 (23%), ID4 (19%), APC (15%), RUX3 (13%), GSTP1 (9%), TP73 (6%), and TIMP3 (4%). RASSFIA1, CCND2, CDH13, CACNA1A*,* HIN-1, sFRP5*,* and ID4 genes that were frequently methylated in OCCA specimens overlapped with those found to be methylated in the OCCA cell lines as shown above. Moreover, the methylation frequencies of RASSFIA*,* CDH13*,* CACNA1A, HIN-1, DKN2B, sFRP5, ID4*,* and ESR were significantly higher among OCCA than those in non-clear-cell types of ovarian carcinoma **(**Table 3).

**Table 2:** Frequencies of promoter methylation which significantly differ in between ovarian clear cell adenocarcinoma and benign endometriotic cysts

	**Percentage (case number)**		
**Gene name**	**Clear cell**	**Benign ovarian cyst**	**p value**
	**n=47**	**N=29**	
RASS328	72% (34)	0% (0)	<0.001
RASS382	77% (36)	0% (0)	<0.001
APC	15% (7)	0% (0)	0.029
CDKN2A	2% (1)	20% (6)	0.006
CDH13	47% (22)	0% (0)	<0.001
SCG148	40% (19)	14% (4)	0.014
SCG409	13% (6)	0% (0)	0.046
SFRP5211	23% (11)	0% (0)	0.004
CCND2220	30% (14)	6% (2)	0.001
CCND2142	51% (24)	24% (7)	0.02
CACNA1A	43% (20)	0% (0)	<0.001

**Table 3:** Frequencies of promoter methylation which differ significantly between ovarian clear cell adnocarcinoma and non-OCCA tissues

	**Percentage (case number/total)**		
**Gene name**	**Clear cell**	**Non-clear cell**	**p value**
	**(n=47)**	**(n=63)**	
RASS328	72% (34)	16% (10)	<0.001
RASS382	77% (36)	17% (11)	<0.001
ESR1	9% (4)	0% (0)	0.018
CDKN2B	28% (13)	5% (3)	0.001
CDKN1B	6% (3)	0% (0)	0.042
CDH13	47% (22)	11% (7)	<0.001
BRCA1	0% (0)	17% (11)	0.002
SCG148	40% (19)	8% (5)	0.001
ID4346	19% (9)	5% (3)	0.016
SFRP382	11% (5)	0% (0)	0.008
SFRP5211	23% (14)	6% (4)	0.01
CACNA1A	43% (20)	11% (7)	<0.001

Endometriosis was reported to be a precursor lesion of OCCA. We next examined methylated genes in OCCA with and without endometriosis. When we compared those genes with methylated promoters in samples of OCCA with and without endometriosis, only CACNA1A had a significantly higher promoter methylation status in OCCA samples without endometriosis (47.2%, 17/36) compared to those associated with endometriosis (0%, 0/8) (*p* = 0.013). And the promoter methylation of CACNA1A was found in benign endometriotic cysts.

Our results indicate that methylation of various genes were specifically related with OCCA instead of the other histologic types of ovarian cancer. And the methylation of CACNA1A gene only participates in the carcinogenesis of OCCA without endometriosis.

### MS-MLPA profiles related to clinical outcome of OCCA patients

**Figure 1:**
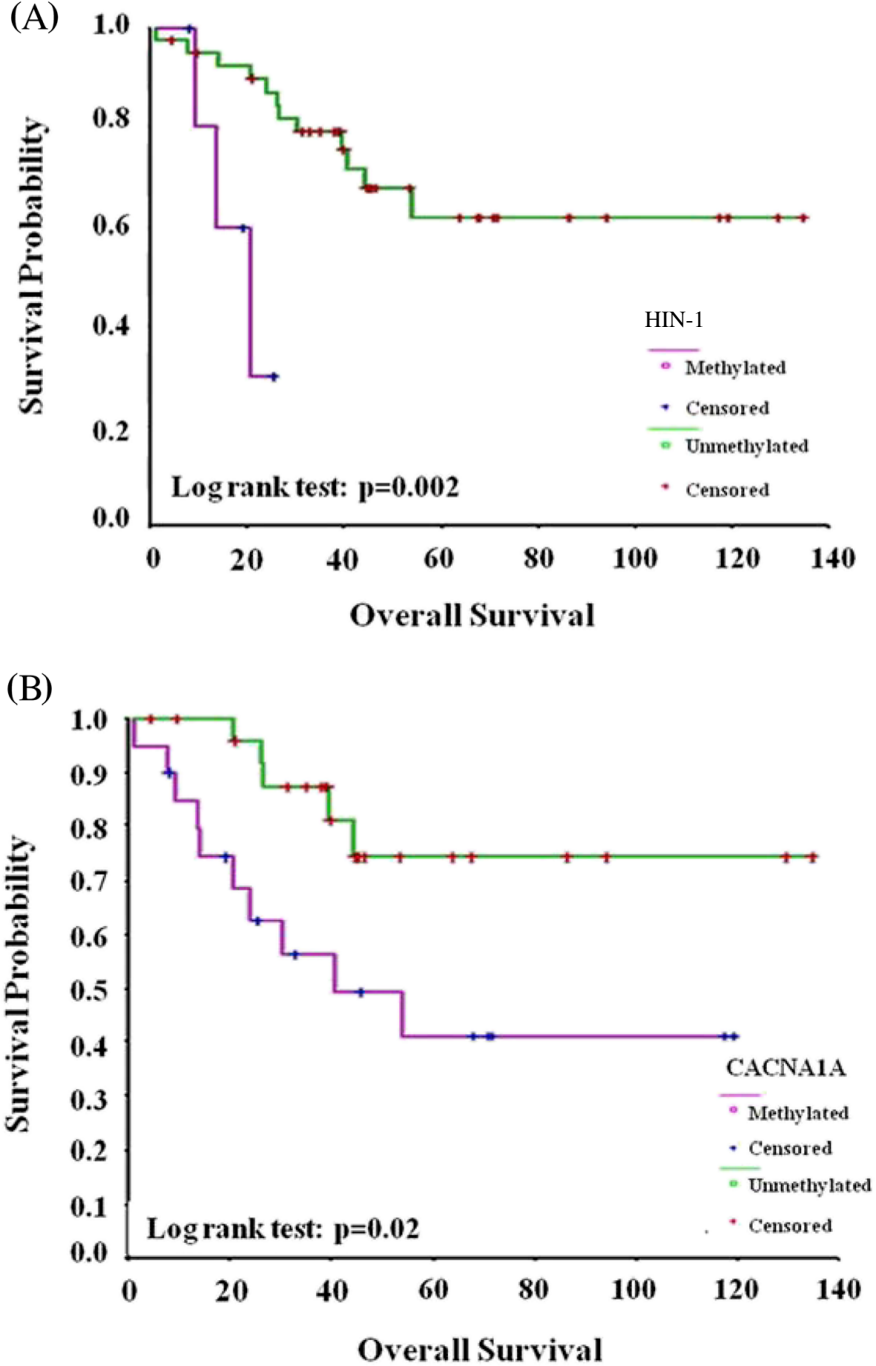
**(A) Overall survival differences in OCCA patients with methylated and unmethylated HIN-1.** (*p* =0.002, by the log-rank test) (**B**) Overall survival differences between OCCA patients with methylated and unmethylated CACNA1A. (*p* =0.02, by the log-rank test).

We then evaluated the correlation between TSG promoter methylation and the progression-free survival (PFS) and overall survival (OS) of OCCA patients. With a median follow-up of 56 months, the expected 5-year PFS and OS for patients with methylated promoters of HIN-1 and CACNA1A genes were significantly worse than those for patients without methylated HIN-1 (28% vs. 54%, *p* = 0.047 for PFS; 30% vs. 62%, *p* = 0.002 for OS, respectively) (Figure 1A) and CACNA1A (30% vs. 63%, *p* = 0.01 for PFS; 40% vs. 75%, *p* = 0.02 for OS) (Figure 1B). Suboptimal surgery, advanced FIGO stages, HIN-1 methylation, CACNA1A methylation, and a higher preoperative CA125 level (> 500 U/ml) were poor prognostic factors for OS of these patients by univariate analysis. Only HIN-1 methylation, (hazard ratio (HR) 13.03, 95% confidence interval (CI) 2.50 ~ 68.58) and CACNA1A methylation (HR 4.30, 95% CI 1.40 ~ 13.27) were independent poor prognostic factors by the multivariate analysis (Figure 1).

### Validation of promoter methylation of the HIN-1 gene with MS-PCR and sodium bisulfite sequencing in samples of OCCA cell lines

**Figure 2:**
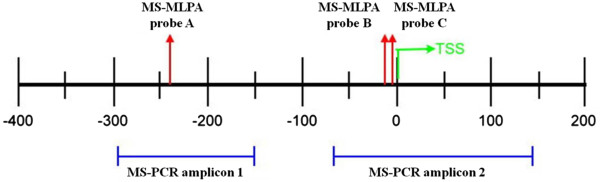
MS-MLPA and MS-PCR probes and amplicon locations of the HIN-1 gene promoter.

**Figure 3:**
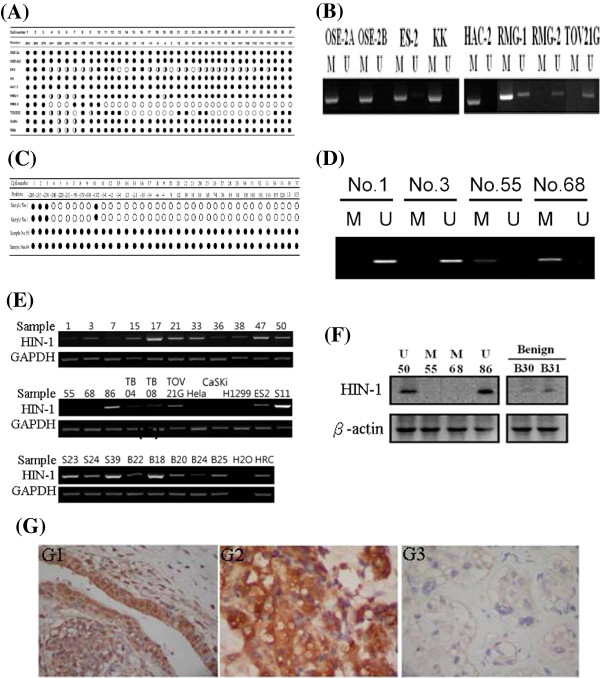
**(A) Summary of bisulphite sequence results of HIN-1 in OCCA cell lines.** The specific CpG sites detected by MS-PCR were almost completely in accordance with bisulphite sequencing of HIN-1. (**B**) Promoter methylation of HIN-1 in OCCA cell lines detected by MS-PCR. (**C**) Summary of bisulphite sequence results of HIN-1 in OCCA tissues. (**D**) Promoter methylation of HIN-1 in OCCA cell lines detected by MS-PCR. (**E**) The mRNA expression of HIN-1 correlated well with the methylation status in OCCA cell lines and cancerous tissues, ovarian serous cancers, and benign endometriotic cysts. (**F**) Representative figures between the methylation status and protein expression of HIN-1 by Western blot analysis. Samples 55 and 68 were OCCA tissues with HIN-1 methylation. Samples 50, 86, 04, and 08 were OCCA tissues without methylation. Samples B30 and B31 were benign endometriotic cysts. (**G**) Immunohistochemical staining of HIN-1. E1: Endometriotic cyst tissue (40x10), E2: HIN-1 overexpression from OCCA (sample 68 with unmethylated HIN-1, 40x10), E3: loss of HIN-1 expression from OCCA (sample 50 with methylated HIN-1, 40x10).

Bisulphite sequencing of CpGs of six OCCA cell lines (ES-2, KK, HAC-2, RMG-1, RMG-2, and TOV21G) and OSE2A and OSE2B was analyzed. The MS-MLPA and MS-PCR probes and amplicon locations within the HIN-1 gene promoter are shown in Figure 2. A summary of the bisulphite sequencing results of HIN-1 in OCCA cell lines is given in Figure 3A. All CpGs were completely methylated in the KK and HAC-2 cell lines, were almost completely methylated in the RMG-1 cell line, and were partially methylated in the ES-2 and TOV21G cell lines. However, almost complete unmethylatation of the RMG-2 cell line was noted. Methylation of specific CpG sites in OCCA cell lines detected by the MS-PCR was in accordance with bisulphite sequencing of the promoter region of *HIN-1*. A representative figure of promoter methylation of *HIN-1* in OCCA cell lines detected by MS-PCR is shown in Figure 3B. Similar findings were found in OCCA tissues. A summary of the bisulphite sequencing results of HIN-1 in OCCA tissues is given in Figure 3C. A representative figure of promoter methylation of *HIN-1* in OCCA tissues detected by MS-PCR is shown in Figure 3D. The MS-PCR and MS-MLPA analyses of promoter methylation of the HIN-1 gene in 47 OCCA tissues showed good correlations (κ = 0.782).

### Promoter methylation and loss of expression of HIN-1 in OCCA tissues

We then checked the impact of promoter hypermethylation on the expression of the *HIN-1* gene. The mRNA level of HIN-1 had a reversed correlation with its methylation status in OCCA cell lines, cancerous tissues, and benign endometriotic cysts (Figure 3E). The representative figures of Western blot analysis of HIN-1 protein in samples with a methylated or unmethylated HIN-1 promoter are shown in Figure 3F. The protein level of HIN-1 also had reversely correlated with the promoter methylation status in OCCA tissues. HIN-1 protein levels in OCCA samples were further checked by immunohistochemistry (Figure 3G). Reduced or absent expression of HIN-1 was observed in 14 (42%) of 33 OCCA specimens. The percentage of HIN-1-expressing in the HIN-1-promoter unmethylated OCCA were significantly higher than that in those on the HIN-1-promoter methylated OCCA (74% (14/19) vs. 32% (5/14), p = 0.029 chi-square test).

Our results indicate that both of the mRNA and protein levels of HIN-1 were lower in specimens with a methylated promoter as compared to those with an unmethylated promoter.

### HIN-1 expression in *HIN-1*-methylated OCCA cancer cells could be restored by the demethylating agent

**Figure 4:**
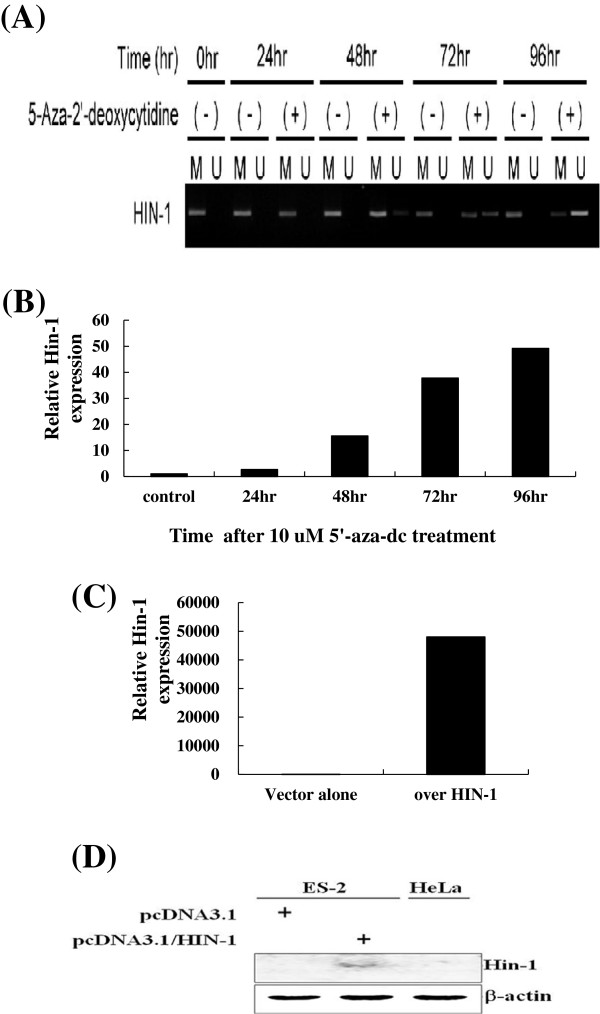
**(A) The representative figure of mRNA expression of HIN-1 in KK cells treated with 5-aza-2-dC at different time intervals.** (**B**) mRNA expression of HIN-1 in KK cells treated with 5-aza-2-dC. After normalization, the relative HIN-1 mRNA level significantly increased in KK cells by 5-aza-2-dC. (**C**) mRNA levels of ES-2 transfectants. mRNA levels of ES-2 cells transfected with HIN-1 were significantly higher than those of ES-2 transfected with EV (*p* < 0.0001) (D) HIN-1 protein expression levels of ES-2 transfectants. Protein levels of ES-2 cells transfected with HIN-1 were significantly higher than those of ES-2 transfected with EV (*p* < 0.0001).

To verify the role of DNA methylation and the level of expression of the HIN-1 gene, fully methylated KK cancer cells were treated with the demethylating agent, 5-aza-2-dC at 0, 24, 48, 72, 96 hours, and the density of unmethylated band of HIN-1 gene was gradually reversed after 48 hoursdetected by MS-PCRe shown in Figure 4A. After normalization, relative HIN-1 mRNA levels in KK cells significantly increased after treatment with 5-aza-2-dC when the duration extended (Figure 4B), which is correlated with HIN-1 DNA methylation (Figure 4A).

### Overexpression of HIN-1 enhanced ES-2 cells sensitivity to paclitaxel

**Figure 5:**
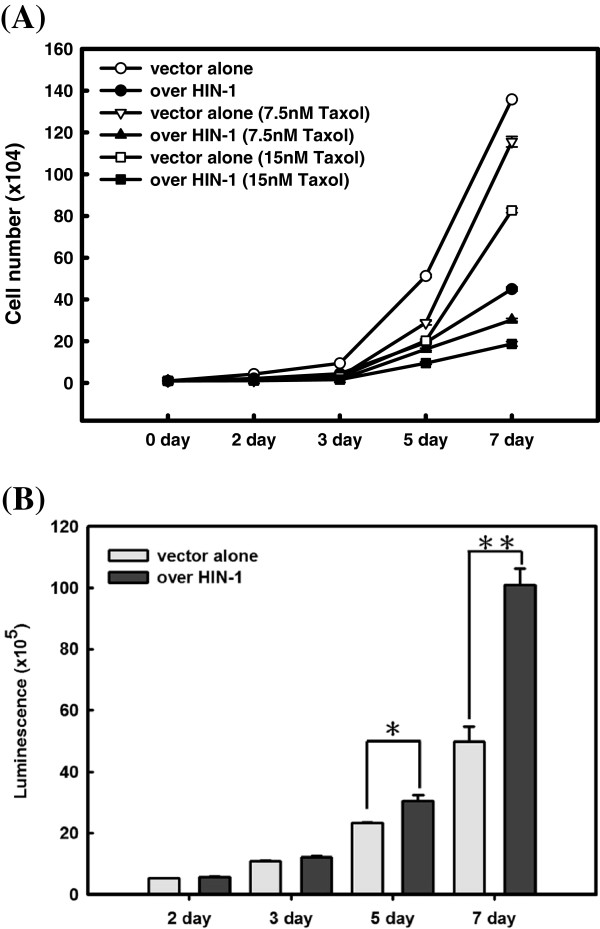
**(A) Cell growth assays of various ES-2 transfectants treated with paclitaxel.** Paclitaxel inhibited cell growth of ES-2 transfectants in a dose-dependent manner. In addition, the inhibitory effects of paclitaxel on cell growth were higher in the ES-2 HIN-1 transfectant than in EV-transfected ES-2 cells (circles, *p* = 0.0001; triangles, *p* = 0.0005; squares, *p* = 0.0008). (**B**) Caspase activities of various ES-2 transfectants. Caspase-3 activities of ES-2 HIN-1 transfectants were higher compared to those of EV-transfected ES-2 cells (for 5 days * *p* < 0.05; for 7 days, ** *p* < 0.001).

We then evaluated cell growth and the viability of ES-2 cells stably transfected with HIN-1 full-length cDNA or an empty vector (EV) as ES-2-HIN-1, and ES-2-PCDNA3.1 cells. mRNA and protein levels of HIN-1 were significantly higher in ES-2-HIN-1 cells than in ES-2-PCDNA3.1 cells (*p* < 0.0001) (Figure 4C and D). In addition, ES-2-HIN-1 cells showed significantly slower growth than ES-2-PCDNA3.1 cells (*p* < 0.0001) (Figure 5A). Furthermore, ES-2-HIN-1 cells had significantly slower growth rate and increasing caspase-3 enzymatic activities compared to those detected in ES-2-PCDNA3.1 cells, when treated with paclitaxel (*p* < 0.01, onw-way ANOVA) (Figure 4A and B).

**Figure 6:**
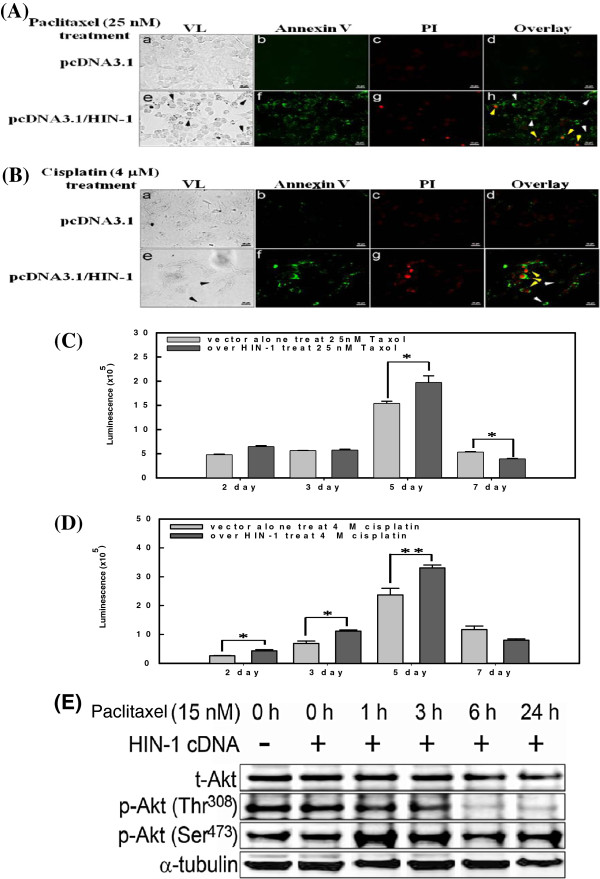
**(A) Representative figures of apoptotic assays of various ES-2 transfectants treated with paclitaxel.** (**B**) Representative figures of apoptotic assays of various ES-2 transfectants treated with cisplatin. ES2 cells without (a-d) or with (e-h) HIN-1 overexpression. The induction of apoptosis is indicated as black arrows in VL (panel e) and white arrows in the overlay (panel h). Late apoptosis is represented as yellow arrows in panel h. VL, visible light; PI, propidium iodide. Bar, 50 μm. (**C**) Caspase-3 activities of various ES-2 transfectants treated with paclitaxel. Increasing caspase-3 activities were shown by ES-2 HIN-1 transfectants compared to EV-transfected ES-2 cells, when treated with paclitaxel. (* *p* < 0.05) (**D**) Caspase-3 activities of various ES-2 transfectants treated with cisplatin. Increasing caspase-3 activities were shown by ES-2 HIN-1 transfectants compared to EV-transfected ES-2 cells, when treated with cisplatin (* *p* < 0.05, ** *p* < 0.001). (**E**) Akt Phosphorylation in HIN-1-overexpressed ES-2 cells. Western blots for phospho-Akt (p-Akt) and total-Akt (t-Akt) were prepared from ES-2 cells treated with 15 nM paclitaxel for 0 h, 1 h, 3 h, 6 h, or 24 h. A marked decrease of p-Akt (Thr^308^) was observed. α-tubulin was as a loading control.

To study the possible role of HIN-1 in the sensitivity of OCCA cells to paclitaxel, ES-2-HIN-1 cells were treated with various concentrations of paclitaxel. As shown in Figure 6A, paclitaxel could induce higher percentages of apoptosis in ES-2-HIN-1 tumor cells than those of vector alone ES-2 tumor cells. Regardless being treated with paclitaxel (Figure 6A) or cisplatin (Figure 6B), ES-2-HIN-1 cells had higher numbers of Annexin-V-positive cells than ES-2-PCDNA3.1 cells (*p* < 0.001). In addition, caspase-3 activities detected in ES-2-HIN-1 cells were also significantly higher than those detected in ES-2-PCDNA3.1 cells, regardless of whether they were treated with paclitaxel (*p* < 0.01, Figure 6C) or cisplatin (*p* < 0.01, Figure 6D).

We further evaluated if the HIN-1 would influence the phosphorylation of Akt. As shown in Figure 6E, the paclitaxel could reduce the phosphorylation of Akt at thr308, but not at Ser473 in ES-2-HIN-1 cells.

Our results indicate that restoration of HIN-1 can enhance the susceptibility of cytotoxic drugs and increase the apoptosis-related enzyme activities in H1N-1-transfected cancer cells by influencing the Akt phosphorylation.

## Discussion

The present study evaluated the application of a multiplex methylation technique to analyze promoter methylation of TSGs in OCCAs. When we tested OCCA cell lines and cancerous tissue specimens, and compared them with ovarian serous carcinomas, different methylation profiles of TSG promoters in OCCA were noted. We then tested promoter methylation frequencies of targeted TSGs in benign endometriotic cysts, which are regarded as precursor lesions in OCCA and compared those with OCCA and non-OCCA tissues. The percentages of promoter hypermethylation of RASSFIA, CCND2, CDH13, CACNA1A, HIN-1, sFRP5, ID4, APC, GSTP1, and TP73 were found from 77% to 6% of OCCAs, respectively, whereas there was little promoter methylation in benign ovarian endometriotic cysts.

The methylation frequencies of RASSFIA*,* CDH13*,* CACNA1A, HIN-1, DKN2B, sFRP5, ID4, and ESR were significantly higher in the clear-cell type of ovarian carcinoma than in the non-clear-cell type. In this study, we included genes with crucial roles in DNA repair and cell cycle control, transcriptional regulation, cell differentiation and proliferation, cell adhesion, DNA damage and apoptosis, and detoxification. Our data are in accordance with previous reports showing altered methylation patterns of a panel of genes in ovarian cancer, including genes encoding RASSFIA, HIN-1, APC, BRCA1, CASP8, CDH13, CDKN2A, CDKN2B, GSTP1, MLH1, PTEN, RASSF1, TIMP3, sFRP4, sFRP5, CCND2 and TP73, which had various promoter methylation extents above the 30% threshold [9,22,23].

Promoter methylation of CACNA1A is associated with OCCA. Our study identified eight candidate genes with promoter methylation in OCCA including CACNA1A and HIN-1, which were significantly higher than those of the non-clear-cell type of ovarian carcinoma. This is the first report of promoter methylation of CACNA1A associated with the carcinogenesis of OCCA. In contrast, promoter methylation of *CACNA1A* was not found in benign endometriotic cysts, and was at a low frequency in ovarian serous carcinoma in this study. Methylation of CACNA1A is involved in the carcinogenesis of OCCA without endometriosis. Promoter methylation of HIN-1 and CACNA1A represents two novel epigenetic events associated with outcomes of patients with OCCA. The biological significance of the methylation of these two aberrant genes with carcinogenesis of OCCA deserves further investigation.

The MS-MLPA offered a good approach to test and improve the histopathologic stratification and prognostic prediction of cancers. Castro et al. reported that MS-MLPA can be used to classify certain types of lung cancers and predict clinical outcomes of patients, and offers an alternative strategy for clinically managing such patients [19]. Among the high-throughput techniques available today for epigenetic alteration assessment, the CpG array represents the main comprehensive platform which has been applied to identify methylation candidates in ovarian cancer [24,25]. However, the CpG array is not feasible for DNA extracted from paraffin-embedded tissues. Advantages of the MS-MLPA technique applied in this study include several aspects such as allowing the screening of promoter methylation of multiple genes in one experiment using a low amount of DNA (around 150 ng), being feasible for using DNA extracted from paraffin-embedded tissues (near half in our OCCA samples), providing semiquantitative data, and requiring only standard laboratory equipments.Advantages of the MS-MLPA using a methylation-sensitive digestion technique, compared to MS-PCR, can omit the potentially troublesome bisulfite conversion of unmethylated cytosines required by the MS-PCR. Methylation indices for the majority of the probes were consistent and reproducible. Variations in methylation ratios obtained for each probe revealed inter-assay reproducibility reliable enough for clinical practice.

To further validate the promoter methylation of HIN-1, two specific regions of promoter methylation of the HIN-1, MS-PCR-1 and MS-PCR-2 amplicons were designed in this survey. These two amplicons overlapped specific sites designed for MS-MLPA of HIN-1. We further used MS-PCR and sodium bisulfite sequencing to validate the specific sites of methylation in OCCA cell lines and cancerous tissues obtained from MS-MLPA. A good correlation of the methylation status between MS-MLPA and MS-PCR was found. We also identified that methylation of HIN-1 downregulated the expression of HIN-1 in OCCA cell lines and cancerous tissues according to RT-PCR, Western blot, and IHC analyses. In addition, HIN-1 expression in the KK cell line (fully methylated HIN-1 gene) was restored by the demethylation reagent, 5-aza-2-dC.

Identifying different methylation profiles in OCCA cell lines provided initial insights into the potential impacts of these candidate genes on OCCA patients. In our series, results for OCCA tissues obtained by MS-MLPA concurred with those of OCCA cell lines, which supported the cancer specificity of the methylated candidates. Our results are also in line with previous reports describing methylation of some candidates in ovarian cancer, such as RASSF1A, CCDN2, CDH13, CACNA1A, HIN-1, SFRP5, ID4, APC , RUX3, GSTP1, TP73, and TIMP3. It is important to be aware that aberrant methylation needs to meet the cutoff ratio of ≥ 30% set by the mathematical algorithm designed to distinguish legitimate methylation peaks. Near half of the OCCA samples in our study were paraffin-embedded tissues, which were microdissected, to prevent contamination by normal (U) cells in the tumor samples. In contrast, detection of an unmethylated promoter next to methylated sequences is usually disregarded as originating from normal tissue. MS-MLPA is only based on one or two CpG sites compared to an average of four to six CpG sites in MS-PCR assays. Since only a small region of the promoter is analyzed with the MS-MLPA method, the methylation of nearby CpG islands cannot be excluded and should be validated by MS-PCR and sodium bisulfite sequencing.

DNA hypermethylation of TSGs plays an important role in ovarian carcinogenesis [9,20,21]. Our data support the concept that promoter hypermethylation is a common mechanism involved in ovarian carcinogenesis, and 11 target genes (RASSF1A, CCDN2, CDH13, CACNA1A, HIN-1, sFRP5, ID4, APC, RUX3, GSTP1, and TP73) novel to OCCA were identified. These results highlight the importance of epigenetic regulation of different types of ovarian cancer. In particular, promoter methylation of HIN-1 was found to be a independent prognostic factor of OS in OCCA.

HIN-1 encodes a small, 10-kDa secreted protein, secretoglobin 3A1, which belongs to the secretoglobin family [24]. Mice with homozygous deletion of HIN-1 are predisposed to develop spontaneous malignancies [26]. Recent reports showed that HIN-1 expression is downregulated in the majority of lung, breast, prostate, pancreatic, colorectal, testicular, and nasopharyngeal cancers, and this downregulation is associated with hypermethylation of the HIN-1 promoter [27-30]. Thus, silencing of HIN-1 expression by promoter methylation is an early and frequent event in multiple human cancer types, functionally relevant to tumorigenesis [27]. Together with the *in vitro* data on growth inhibition and Akt activation in breast cancer, these results suggest that HIN-1 is a candidate tumor suppressor gene [31]^.^ We demonstrated herein that the frequently of HIN-1 promoter hypermethylation occurs in OCCA but less often in non-OCCA-type epithelial ovarian cancer, suggesting that this event may also play a role in the development of a subgroup of these tumors. We further confirmed that HIN-1 is downregulated by promoter methylation and functions as a tumor suppressor gene through inhibiting cell growth and inducing apoptosis in OCCA cells. Furthermore, overexpression of HIN-1 enhanced ES2 cell sensitivity to paclitaxel and cisplatin through significantly inhibiting cell growth and increasing early and late apoptosis, which supports the results of HIN-1 promoter methylation with downregulation of HIN-1 expression causing poor survival outcomes in OCCA patients. From the results of reducing phosphorylated Akt at thr308, ectopic expression of the HIN-1 gene in OCCA cells increased paclitaxel sensitivity which is possibly through the Akt pathway.

OCCA is usually more resistant to systemic chemotherapy than other types and has a poorer prognosis [3-5]. Almost all OCCA patients undergo subsequent adjuvant chemotherapy, with a combination of taxane and platinum compounds which are the most frequently applied regimens. The lack of effective chemotherapy for recurrent OCCA after frontline treatment is another important clinical problem. Therefore, to improve the survival of patients with OCCA, the development of novel treatment strategies in the setting of both first-line and salvage treatments of recurrent disease is urgently needed. If the sensitivity to taxane-based chemotherapy can be enhanced, it would represent improved clinical management of this disease. In the present study, whether methylation of HIN-1 or CACNA1A gene could predict survival and also act as a prognostic indicator when treating OCCA patients. Our study provides novel and useful information for planning treatment strategies of OCCA. In this study, overexpression of HIN-1 enhanced ES2 cell sensitivity to paclitaxel and cisplatin through inhibiting cell growth and enhancing apoptosis. Restoration of the function of HIN-1 would be an important step to develop new treatment strategies with demethylating agents or histone deacetylating inhibitors for OCCA patients with HIN-1 methylation. The underlying mechanisms and pathways regulating the HIN-1 gene in OCCA urgently need to be explored.

Recently, CACNA1A was identified as a novel tumor suppressor candidate, the promoter of which is methylated in lung and prostate tumors [19-32]. *CACNA1A* was implicated in diseases of the brain, regulated by HIF-2α [33]. Anglesio et al. pointed out the importance of IL6-STAT3-HIF signaling and the promising response to the angiogenesis inhibitor, sunitinib, in OCCA patients [33], and suggested that more-extensive clinical trials with sunitinib in OCCA were warranted. Intriguingly, *HIN-1* was also identified as a direct HIF-2α-targeted gene, and it was demonstrated that HIF-2α regulates HIN-1 expression and tumor formation in human ras^G12D^-driven NSCLC cells [34]. Akt pathway activity repressed by HIN-1 was enhanced in HIF-2α-deficient human NSCLC cells and xenografts [35]. The role of HIF-2α regulation in OCCA needs to be explored.

## Competing interests

The authors declare that they have no conflicts of interest.

## Authors’ contributions

CMH applied for the grant, and conducted the study, and participated in its design and coordination, and draft manuscript. CJH helped molecular analyses and interpretation. CYH performed statistical analysis. YYW performed the pathologic analysis and interpretation. SFC and WFC participated in designing and coordinating of the study and helped draft the manuscript. All authors read and approved the final manuscript.
